# Mapping Quantitative Trait Loci for Tolerance to *Pythium irregulare* in Soybean (*Glycine max* L.)

**DOI:** 10.1534/g3.118.200368

**Published:** 2018-08-22

**Authors:** Feng Lin, Shabir H. Wani, Paul J. Collins, Zixiang Wen, Cuihua Gu, Martin I. Chilvers, Dechun Wang

**Affiliations:** * Department of Plant, Soil and Microbial Sciences, Michigan State University, East Lansing-48824, Michigan; † Mountain Research Centre for Field Crops, Khudwani, Anantnag-192 101, Sher-e-Kashmir University of Agricultural Sciences and Technology of Kashmir, J&K, India

**Keywords:** *Pythium irregulare*, Quantitative Trait Loci Mapping, Single Nucleotide Polymorphism

## Abstract

*Pythium* root rot is one of the significant diseases of soybean (*Glycine max* (L.) Merr.) in the United States. The causal agent of the disease is a soil-borne oomycete pathogen *Pythium irregulare*, the most prevalent and aggressive species of *Pythium* in North Central United States. However, few studies have been conducted in soybean for the identification of quantitative trait loci (QTL) for tolerance to *P. irregulare*. In this study, two recombinant inbred line (RIL) populations (designated as POP1 and POP2) were challenged with *P. irregulare* (isolate CMISO2-5-14) in a greenhouse assay. POP1 and POP2 were derived from ‘E09014’ × ‘E05226-T’ and ‘E05226-T’ × ‘E09088’, and contained 113 and 79 lines, respectively. Parental tests indicated that ‘E05226-T’ and ‘E09014’ were more tolerant than ‘E09088’, while ‘E09088’ was highly susceptible to the pathogen. The disease indices, root weight of inoculation (RWI) and ratio of root weight (RRW) of both populations showed near normal distributions, with transgressive segregation, suggesting the involvement of multiple QTL from both parents contributed to the tolerance. All the lines were genotyped using Illumina Infinium BARCSoySNP6K iSelect BeadChip and yielded 1373 and 1384 polymorphic markers for POP1 and POP2, respectively. Notably, despite high density, polymorphic markers coverage was incomplete in some genomic regions. As such, 28 and 37 linkage groups were obtained in POP1 and POP2, respectively corresponding to the 20 soybean chromosomes. Using RRW, one QTL was identified in POP1 on Chromosome 20 that explained 12.7–13.3% of phenotypic variation. The desirable allele of this QTL was from ‘E05226-T’. Another QTL was found in POP2 on Chromosome 11. It explained 15.4% of the phenotypic variation and the desirable allele was from ‘E09088’. However, no QTL were identified using RWI in either population. These results supported that RRW was more suitable to be used to evaluate *P. irregulare* tolerance in soybean.

Soybean (*Glycine max* (L.) Merr) is an essential oil and protein crop grown worldwide including the United States. Oomycetes of the genus *Pythium* are the principal causes of seed and seedling rot of soybean, which are frequently grown in rotation in the Midwestern United States (Radmer *et al.* 2017, Ellis *et al.* 2013). *Pythium* includes more than 130 documented species and has global distribution (Dick 1990). At least 27 species of *Pythium* host soybean, and *P. irregulare* has been identified to be one of the most prevalent and aggressive *Pythium* species in the United States (Zitnick-Anderson *et al.* 2014, Radmer *et al.* 2017). Hence, root rot caused by *P. irregulare* is one of the most serious root diseases affecting soybean.

*P. irregulare* prefers cold, moist soil conditions and is often the first pathogen to cause diseases during the growing season (Rojas *et al.* 2017). *Pythium* species have been shown to be virulent across a wide range of temperatures (Matthiesen *et al.* 2016, Wrather and Koenning 2009, Wei *et al.* 2010). However, *P. irregulare* showed increased virulence at temperatures between 4° and 12° (Hendrix and Campbell 1973, Wei *et al.* 2010).

Management of soybean seed and seedling rot caused by *Pythium* spp. includes seed treatment with fungicides, such as metalaxyl and mefenoxam (Radmer *et al.* 2017) and other practices including crop rotation and field tillage for proper drainage (Stasko *et al.* 2016). However, the effect of seed treatment with fungicide on seed and emerging seedling lasts only for a limited time (one to two weeks after planting), hence cannot effectively protect the developing root (Paulsrud and Montgomery 2005). Host plant resistance has been demonstrated to be the most cost-effective strategy to combat soybean diseases and insects such as soybean cyst nematode (Cook *et al.* 2012), *Phytophthora* root rot (Lin *et al.* 2013), and soybean aphid (Zhang *et al.* 2017). For *Pythium* disease resistance, although not commonly deployed, host resistance genes have been described to be an optimum option. (Rupe *et al.* 2011, Nanayakkara *et al.*, 2002, Ellis *et al.* 2013, Stasko *et al.* 2016, Urrea *et al.* 2017).

Two types of resistance have currently been reported for resistance to *Pythium* spp. in soybean, including *Rpa1* which was suggested to be an *R*-gene type of vertical resistance to *P. aphanidermatum* (Rosso *et al.* 2008), and many quantitative trait loci (QTL) that contribute to tolerance (or partial resistance). For *P. irregulare*, QTL have been identified on eight soybean chromosomes (Ellis *et al.* 2013, Stasko *et al.* 2016). However, each QTL explained only a small percentage (8–20%) of the phenotypic variation, suggesting a complex mechanism of tolerance.

‘E09014’, ‘E05226-T’, and ‘E09088’ are conventional soybean genotypes developed at Michigan State University. ‘E05226-T’ was developed from ‘Vinton81’ × ‘Syngenta S19-90’. ‘E09014’ was developed from a cross between ‘A00-711003’ and ‘AxN-1-55’, which was from ‘Asgrow A2506’ × ‘Syngenta S19-90’. ‘E09088’ was from ‘LD01-7323’ and ‘Skylla’, which was developed from ‘Dairyland 217’ × ‘Syngenta S19-90’. Two recombinant inbred line (RIL) populations have been made by crossing ‘E05226-T’ with ‘E09014’ and ‘E09088’ for identifying resistance QTL to white mold disease (caused by *Sclerotinia sclerotiorum*). Yet later tests showed that these genotypes also carried variable levels of tolerance to *P. irregulare*. which is an aggressive and widely distributed pathogen with few studies conducted on resistance to. (Rojas *et al.* 2017). The objectives of this study were to i) establish high resolution genetic linkage maps using high density single nucleotide polymorphism (SNP) markers across the soybean genome for both RIL populations, ii) Dissect the genetic components of resistance through genome-wide QTL mapping, and iii) Identify molecular markers that could be used for marker assisted selection for *P. irregulare* resistance in soybean.

## Materials and Methods

### Plant materials

Two recombinant inbred line (RIL) populations were created from ‘E09014’ × ‘E05226-T’ (designated as POP1) and ‘E05226-T’ × ‘E09088’ (designated as POP2). POP1 contained 113 F_4:7_ RILs, 87 of which were genotyped in the F_4:5_ generation to construct a linkage map, whereas the other 26 were genotyped in the F_4:7_ generation for further dissection of QTL. For POP2, 88 F_4_-derived lines were used, with 59 lines genotyped at F_4:5_ for linkage map, and 29 genotyped at F_4:7_. However, 9 F_4:5_ lines from POP2 failed to produce enough seeds for disease evaluation at F_4:7_. Thus, a total of 79 F_4:7_ lines were used for QTL mapping.

### Preparation of Inoculum

*P. irregulare* isolate CMISO2-5-14 (Rojas *et al.* 2016) was used in this study. Prior to use, the isolate was maintained on a Potato Carrot Agar (PCA) slant at 15° in a glass tube. The isolate was first grown for 7-8 days on Petri plates containing corn meal agar medium and then transferred to a rice medium for increase. The rice medium was prepared by adding 300 ml of double-distilled water to 700 g of brown rice (parboiled) grains in an autoclave-safe plastic bag and autoclaved twice for 4 hr at 121°, with 24 hr between the two autoclaving operations. On the third day, when the rice medium cooled down to room temperature, one Petri plate of a 7-8-day-old *P*. *irregulare* isolate was aseptically transferred to rice medium under a laminar air flow cabinet and incubated for 12-14 days at 25°. To ensure proper colonization, the bags were mixed every alternate day until complete colonization occurred.

### Disease evaluation

The disease evaluation was performed at Michigan State University (MSU) greenhouse facilities, with temperature set at 20 - 22°. All lines of POP1 and POP2 were evaluated in a randomized complete-block design, with the three parental lines used as controls. For each line, 6 seeds were used for each replicate. A total of four replicates were used for inoculation for both POP1 and POP2, and three and four replicates were used for the non-inoculated group of POP1 and POP2, respectively. The number of viable seeds of inoculated materials in each replicate was estimated by the number of emerged seedlings from non-inoculated replicated materials. For ‘E05226-T’ and ‘E09014’, 14 and 10 replicates were for inoculated and non-inoculated treatments, respectively. For ‘E09088’, 10 replicates were inoculated, and 6 replicates were non-inoculated as control.

To start the test, seed starting trays were first filled with medium size vermiculite and soaked in water until the vermiculite was fully saturated. Then two 4cm-deep holes were made in each cell and approximately 2g inoculum (or 8-12 rice grains) was placed at the bottom of each hole for inoculation. A single seed was then planted on the top of the inoculum in each hole and covered with vermiculite. For the non-inoculation group, a single seed was planted at the bottom of each hole right after it was made without placing inoculum in it. Seeds were carefully selected for uniform size and without any damage or disease symptoms before planting.

After planting, seed starting trays were transferred to the greenhouse benches that had been covered with waterproof plastic. Benches were watered until the water height reached the level of the inoculum. After that, benches were watered every other day to maintain the flooding environment until two days before measurement. Fourteen days after planting, the number of plants emerged for each line was recorded, and fresh root weight of each line was measured using an electronic balance (Scout Pro, SP 4001; Ohaus Corp, Pine Brook, NJ). The response of each line in each inoculated replicate was measured from fresh root weight of inoculation (RWI) and ratio of root weight (RRW).

RWI  =  total  fresh  root  weight  in  an  inoculated  replicate/number  of  viable  seeds  (N)  

where, N was estimated as average number of germinated seeds across all non-inoculated replicates. To guarantee the high seed vigor, a cutoff of N ≥ 4 was examined for each non-inoculated replicate. All 192 soybean lines in POP1 and POP2 passed the examination and were used in the following analyses.

RRW =RWI of an inoculated replicate/ mean of fresh root weight of control (RWC) 

where, RWC = total fresh root weight of a non-inoculated replicate / Number of germinated seeds of the replicate

### Sample collection and DNA extraction

For linkage map analysis, 10 young leaves were bulk harvested for each of the 87 F_4:5_ lines of POP1 and 59 F_4:5_ lines of POP2. For genotyping an additional 26 lines of POP1 and 29 lines of POP2, 6 young leaves of each line were bulk harvested at F_4:7_ generation. Genomic DNA of each line was then extracted using a modified CTAB method (Wen *et al.* 2014). Briefly, the bulked leaf tissue was first frozen at -80° for at least 24 hr, freeze-dried, ground with glass beads (Fisher Scientific, Pittsburgh, PA), and DNA concentration of each sample was measured with an ND-1000 Spectrophotometer (NanoDrop Technologies, Inc., Wilmington, DE, USA).

### Construction of linkage map

All samples used in this study were genotyped using Illumina Infinium BARCSoySNP6K iSelect BeadChip genotyping array (Illumina, San Diego, USA) (Song *et al.* 2013). Based on the parental genotypes, SNP markers that were not polymorphic or had missing parental data were removed. After filtering, 1,373 and 1,384 polymorphic markers remained between the two parents for POP1 and POP2, respectively, which were then imported into Joinmap software (v4.0) for linkage analysis (Van Ooijen 2006). Marker order was determined using maximum likelihood algorithm and mapping function was used to determine genetic distance. Linkage groups were then determined using an independence LOD = 4.0 and a max recombination frequency = 0.5. Markers that were not grouped with at least five other markers were excluded. Linkage maps were drawn using MapChart software (Voorrips 2002).

### Statistical and QTL Analyses

The statistical descriptive analysis in this study was performed using SPSS software (IBM SPSS Statistics, IBM Corporation, Chicago, IL). The broad-sense heritability was estimated according to the method described by Fehr (1987). The software QTL Cartographer V2.5 (Wang *et al.* 2012) was used for interval mapping (IM) and composite interval mapping (CIM). Window size was 5cM and the walking speed was 1cM. The threshold of LOD score for statistical significance of QTL effects was determined by 1,000 permutations, and the LOD value corresponding to an experiment-wise Type I error rate of 5% (α = 0.05) was considered the threshold of significance (Churchill and Doerge 1994). The position of QTL was estimated as the point of maximum LOD score in the region under consideration.

### Data Availability

All soybean lines and *Pythium irregulare* isolates are available upon request. The phenotypic data are included in file Supplementry_Datafile_1 (parental lines), Supplementary_Datafile_3 (POP1), and Supplementary_Datafile_5 (POP2). File Supplementary_Datafile_2 and Supplementary_Datafile_4 are genotypic data of POP1 and POP2, respectively, obtained from BARCSoySNP6K chip. ‘a’ represents genotype of ‘E05226-T’, ‘b’ represents the genotype of the other parental line, and ‘h’ represents heterozygous genotype.. Supplemental material available at Figshare: https://figshare.com/articles/Supplementary_figures_and_files/6943328.

## Results

### Response of soybean lines to *P. irregulare*

As expected, ‘E05226-T’ conferred the highest level of tolerance to *P. irregulare* using either RWI (0.164 ± 0.159 for mean ± SD) or RRW (0.181 ± 0.176), followed by ‘E09014’ (0.084 ± 0.090 for RWI and 0.104 ± 0.111 for RRW). The RWI and RRW of ‘E05226-T’ and ‘E09014’ were not significantly different but were both significantly higher than those for ‘E09088’. ‘E09088’ was nearly completely susceptible, with a mean of 0.005 for RWI and 0.005 for RRW. For the non-inoculated control (RWC), the root weight of ‘E05226-T’ (0.903 ± 0.136) was larger than that of ‘E09088’ (0.855 ± 0.074) and ‘E09014’ (0.807 ± 0.152) but no significant difference was observed between them (Table 1).

**Table 1:** Statistics of soybean response to *Pythium irregulare*

	RWI (g) Mean ± SD.	Range	RWC (g) Mean ± SD.	Range	RRW Mean ± SD.	Range
E05226-T	0.164^a^ ± 0.159	0.00 – 0.55	0.903^ab^ ± 0.136	0.78 – 1.20	0.181^a^ ± 0.176	0.00 – 0.61
E09014	0.084^ab^ ± 0.090	0.00 – 0.24	0.807^b^ ± 0.152	0.63 – 1.09	0.104^ab^ ± 0.111	0.00 – 0.30
E09088	0.005^b^ ± 0.015	0.00 – 0.05	0.855^ab^ ± 0.074	0.78 – 0.98	0.005^b^ ± 0.017	0.00 – 0.05
POP1 (E09014 x E05226-T)	0.317^c^ ± 0.154	0.00 – 0.69	0.955^a^ ± 0.149	0.48 – 1.34	0.331^c^ ± 0.158	0.00 – 0.70
POP2 (E05226-T x E09088)	0.157^a^ ± 0.084	0.00 – 0.36	1.083^c^ ± 0.094	0.79 – 1.26	0.144^a^ ± 0.076	0.00 – 0.34

Different letters indicate significant differences at α = 0.05 level.

**Figure 1:**
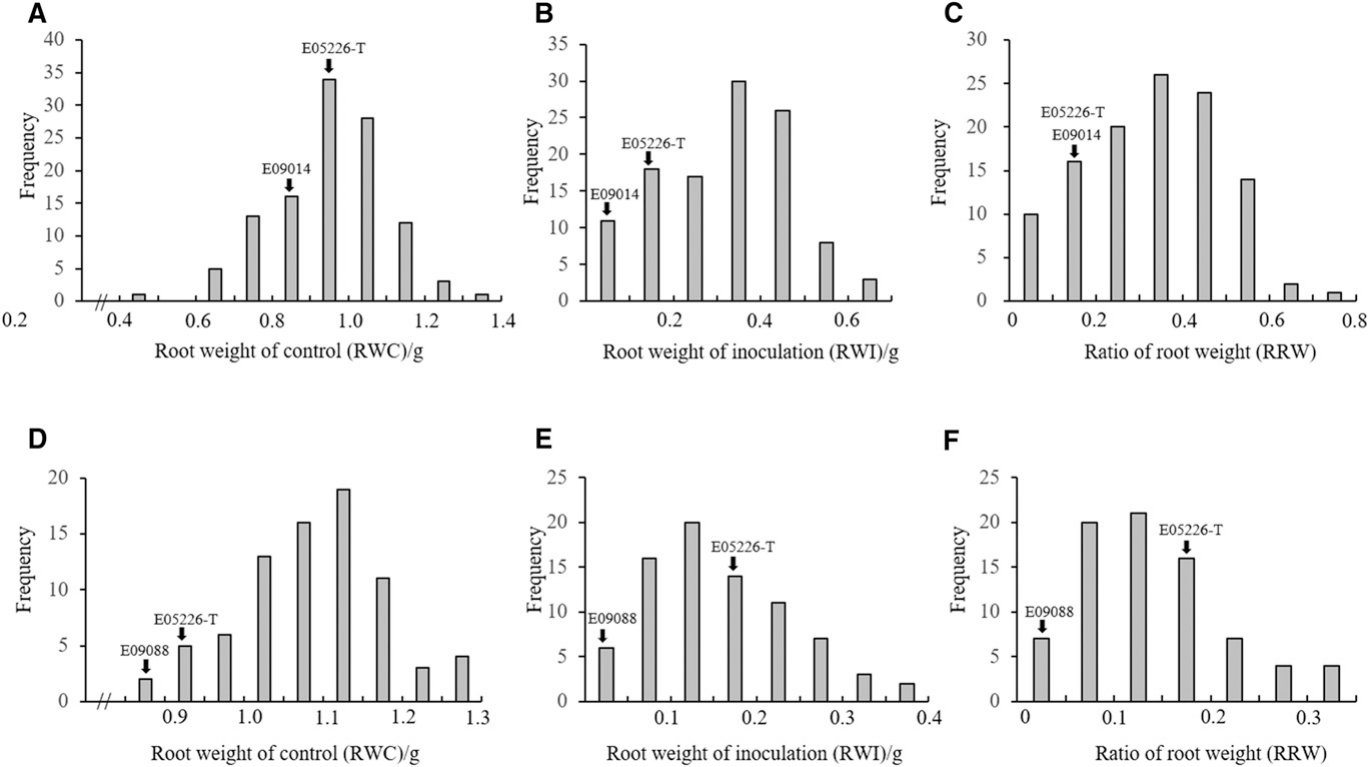
Phenotypic analysis of soybean response to *P. irregulare*. A, B and C: phenotypic analysis soybean lines in POP1. D, E and F: phenotypic analysis of soybean lines in POP2. A and D: fresh root weight of non-inoculated control (RWC). B and E: fresh root weight of inoculation (RWI). C and F: ratio of root weight (RRW). Black arrow indicates the position of parental lines in each population.

RWI and RRW were nearly normally distributed in both populations, suggesting multiple QTL contributing to the tolerance (Figure 1). The tolerance of POP1 was significantly higher than that of POP2 (Table 1). The mean RWI of POP1 was 0.317g, which was significantly higher than that of POP2 (0.157g). However, for the non-inoculated control, the RWC of POP2 (1.083 ± 0.094g) was significantly higher than that of POP1 (0.955 ± 0.149g), resulting in a significantly higher mean RRW for POP1 (0.331 ± 0.158) than POP2 (0.144 ± 0.076) (Table 1). Interestingly, in addition to the transgressive segregation (Figure 1), the mean of POP1 was also significantly higher than those of its parental lines. For POP2, the mean was significantly higher than that of E09088, but it was not significant compared with the mean of E05226-T. This was probably attributable to the higher tolerance of parental lines of POP1. The broad sense heritabilities of RWI for POP1 and POP2 were 0.84 and 0.92, and those of RRW were 0.86 and 0.92, respectively.

### Linkage analysis

The SoySNP6K bead chip contains 5,376 SNP markers which were designed to represent major LD (linkage disequilibrium) blocks across the soybean genome (Akond *et al.* 2013, Wen *et al.* 2014). In this study, a total of 1,384 and 1,373 polymorphic SNP markers were used to develop linkage maps for POP1 and POP2, respectively. Large genomic regions with monomorphic markers were observed, *i.e.*, chromosomes 1, 11 and 14 in POP1, and chromosomes 3, 16 and 20 in POP2 (Supplementary Figure 1). This may be attributable to the similarity of parental lines sharing a common ancestor ‘Syngenta S19-90’ and fixation of desirable alleles for favorable agronomic traits during their development.

Because of the large monomorphic regions, polymorphic markers did not group perfectly into 20 linkage groups, the number of soybean chromosomes. Instead, the 1,384 and 1,373 polymorphic markers were grouped into 28 and 37 linkage groups for POP1 and POP2, respectively (Supplementary Table 1, Supplementary Figure 2, Supplementary Figure 3). In POP1, polymorphic markers on Chromosomes 2, 3, 4, 7, 11, 14, 17 and 18 were separated into two linkage groups. The polymorphic markers of POP1 covered a total of 1,751.52cM and the average marker density ranged from 0.04cM (LG3.2) to 3.15cM (LG4.2) with an average of 1.27cM (Supplementary Table 1). In POP2, two linkage groups were obtained from polymorphic markers on Chromosomes 1, 2, 3, 5, 7, 9, 13, 14 and 16, whereas three linkage groups formed from Chromosomes 4, 8, 17 and 19. The polymorphic markers of POP2 covered a total of 2,093.24cM and the average marker density ranged from 0.26cM (LG8.3) to 6.27cM (LG19.3) with an average of 1.52cM.

### Molecular mapping of QTL

**Figure 2:**
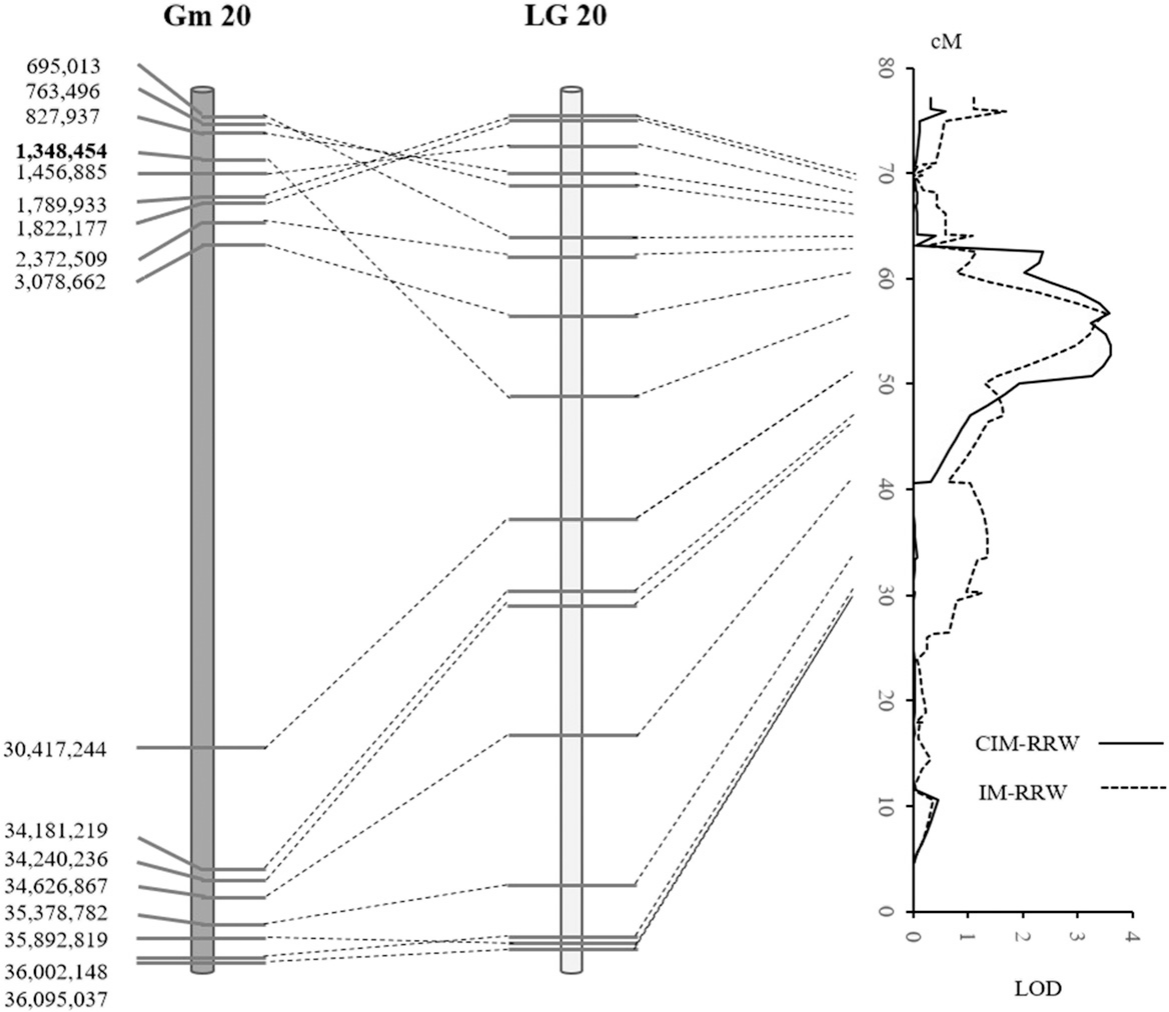
QTL detected in POP1 for tolerance to *P. irregulare*. Left: Physical position of genetic markers flanking QTL based on the Williams82 reference genome (Wm82.a1.v1.1). Bolded markers indicate the closest markers to QTL. Middle and right: Genetic position of flanking markers for QTL.

**Figure 3:**
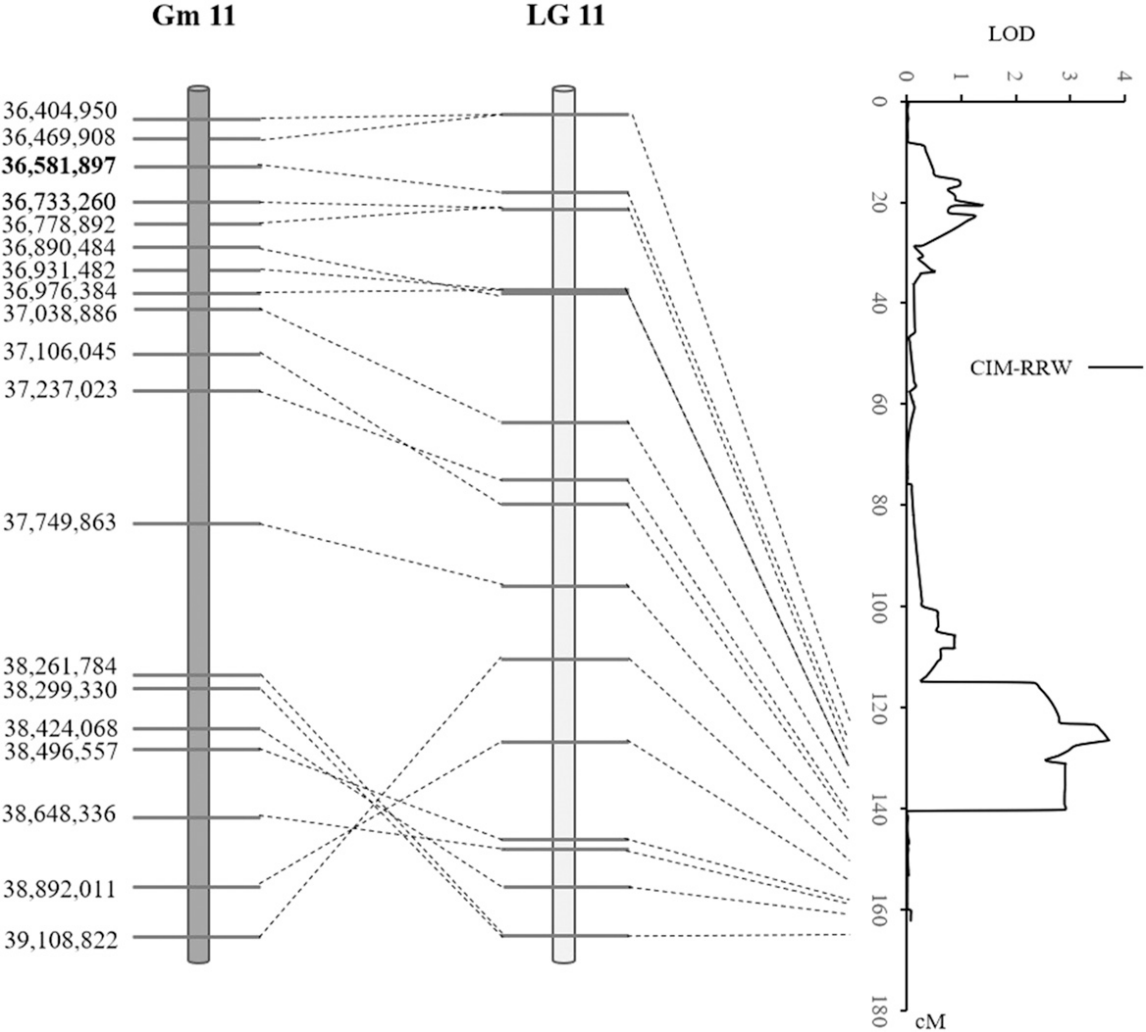
QTL detected in POP2 for tolerance to *P. irregulare*. Left: Physical position of genetic markers flanking QTL based on Williams82 reference genome (Wm82.a1.v1.1). Bolded markers indicate the closest markers to QTL. Middle and right: Genetic position of flanking markers for QTL.

Quantitative trait loci (QTL) were detected in both populations using RRW. In POP1, the first QTL (designated qRRW20) was detected using IM at 56.7cM on chromosome 20 (MLG I) with a LOD of 3.52. The QTL accounted for 13.3% of phenotypic variation and the desirable allele of this QTL was from E05226-T (Table 2). The QTL was flanked by Gm20_1348454_T_G and Gm20_30417244_C_T, with Gm20_1348454_T_G being the closest marker to it (Figure 2). Using CIM, a QTL was detected at 53.7cM on the same chromosome. This QTL was co-located with the first QTL in the same genetic interval and was thus considered the same. In POP2, one QTL (designated qRRW11) was detected using CIM at 126.4cM on Chromosome 11 (MLG B1) with a LOD of 3.70 (Figure 3). The QTL was located between Gm11_33511924_C_T and Gm11_37038886_C_T, and the closest marker to it was Gm11_36581897_A_G. The QTL explained 15.4% of the phenotypic variation and the desirable allele was from E09088 (Table 2). No QTL was detected when using RWI as disease index.

**Table 2:** QTL detected in POP1 and POP2 for resistance to *Pythium irregulare*

Population	Mapping Method	Trait	Chr.	QTL Position (cM)	Nearest Marker	LOD	R^2^	LOD Threshold*	Desirable Allele
POP1 (E09014 x E05226-T)	IM	RRW	Gm20	56.7	Gm20_1348454_T_G	3.52	13.3%	3.50	E05226-T
	CIM	RRW	Gm20	53.7	Gm20_1348454_T_G	3.61	12.7%	3.50	E05226-T
POP2 (E05226-T x E09088)	CIM	RRW	Gm11	126.4	Gm11_36581897_A_G	3.70	15.4%	3.45	E09088

* LOD threshold of significance is determined by permutation tests of 1000 iterations (*P* < 0.05) (Churchill and Doerge 1994).

## Discussion

In this study, we used RWI and RRW as disease index to identify QTL from two soybean RIL populations contributing to the tolerance of *Pythium irregulare* disease. RWI scores the inoculated roots and can reflect the response of soybean root to *Pythium* infection within a line. However, RWI can also reflect the differences of root growth among different soybean lines without considering the non-inoculated control. Therefore, the detection of disease tolerance QTL may be confounded when there’s dramatic differences of root growth among soybean lines. In this study, both POP1 and POP2 showed dramatic segregation for normal root growth (Figure 1A and 1D), which might explain why no QTL was detected. However, RRW considers both inoculation and non-inoculation procedures, and reflects a relative value of disease response to its normal growth, and therefore can detect disease tolerance QTL without being confounded. In our study, two QTL were identified using RRW. Therefore, RRW may outperform RWI for disease evaluation in soybean.

Transgressive segregation was observed for both RIL populations in this study, suggesting that the combination of genetic components from both parents could strengthen the tolerance to disease (Rieseberg *et al.* 1999). In POP1, qRRW20 was detected from E05226-T, but no QTL was detected from E09014. This might be attributable to the small effect of multiple QTL and relatively small population size. In POP2, another QTL qRRW11 was detected from the susceptible parent E09088. The contributions from qRRW20 and qRRW11 may explain the transgressive segregation pattern in POP2.

To develop soybean cultivars resistant to *P. irregulare*, desirable alleles with high heritability are needed. In this study, using either RWI or RRW as a disease index, the resistance to *P. irregulare* was highly genetically controlled. In POP1, the heritability of resistance was more than 80%, and in POP2, the heritability of resistance was more than 90%. The high heritability of resistance to *P. irregulare* can also be supported by other studies (Ellis *et al.* 2013, Stasko *et al.* 2016). Thus, significant improvement could be expected by introgression of the QTL into new soybean cultivars through breeding efforts.

High density genetic maps are critical to improving the accuracy and resolution of QTL mapping, even with low-heritability quantitative traits (Song *et al.* 2016, Cao *et al.* 2017). In our study, BARCSoySNP6K iSelect BeadChip was used to develop high-density linkage maps for the two RIL populations, and to anchor QTL to small genetic regions. For example, the average genetic distance between adjacent markers was 2.24 cM and 1.69 cM in POP1 and POP2, respectively. The genetic distances of nearest markers to qRRW20 and qRRW11 were 2.94 cM and 0.02 cM, respectively, and such narrowly defined genetic regions of QTL can facilitate the use of adjacent markers for marker-assisted selection (MAS) to improve soybean resistance to *P. irregulare*.

Before the current study, significant or putative QTL have been identified on 8 chromosomes using fresh root weight or root rot score as disease index to evaluate resistance to *P. irregulare* (Ellis *et al.*, 2013, Stasko *et al.*, 2016). All these QTL accounted for 5–18% of observed phenotypic variation, which was similar to what was found in this study. Intriguingly, a significant QTL was previously identified on Chromosome 20 using the IM method (Ellis *et al.* 2013), and the QTL (nearest marker BARC-052017-11314) was co-localized with qRRW20. However, this QTL was not detected using CIM method (Ellis *et al.* 2013).

Two types of resistance have currently been discovered for resistance to *Pythium* spp. in soybean, including R-type of resistance (Rosso *et al.* 2008), and quantitative resistance or tolerance (Ellis *et al.*, 2013, Stasko *et al.*, 2016, Urrea *et al.*, 2017). Using a similar hypocotyl inoculation assay and evaluation standards as for *Phytophthora sojae* (Dorrance *et al.* 2004), an R-type resistance gene, *Rpa1* (for *resistance to P. aphanidermatum-1*), was dissected and mapped to MLG F (Chromosome 13) from a resistant soybean cultivar ‘Archer’ (Rosso *et al.* 2008). However, R-type resistance has rarely been studied for *Pythium* spp., and quantitative resistance may be expected because of the wide host range of the pathogen (Urrea *et al.* 2017). Continuous efforts will be required to identify additional resistance sources to provide more durable and effective resistance to protect future soybean cultivars from *Pythium* disease.
